# The wide spectrum high biocidal potency of Bioxy formulation when dissolved in water at different concentrations

**DOI:** 10.1371/journal.pone.0172224

**Published:** 2017-02-16

**Authors:** Dori Dagher, Ken Ungar, Richard Robison, Fadi Dagher

**Affiliations:** 1 Bioxy AFD Inc. and Atomes F.D. Inc., Ville Saint-Laurent, Quebec, Canada; 2 Brigham Young University, Provo, Utah, United States of America; Natural Environment Research Council, UNITED KINGDOM

## Abstract

Traditional surface disinfectants that have long been applied in medicine, animal husbandry, manufacturing and institutions are inconvenient at best and dangerous at worst. Moreover, some of these substances have adverse environmental impacts: for example, quaternary ammonium compounds (“quats”) are reproductive toxicants in both fish and mammals. Halogens are corrosive both to metals and living tissues, are highly reactive, can be readily neutralized by metals, and react with organic matter to form toxic, persistent by-products such as dioxins and furans. Aldehydes may be carcinogenic to both human and animals upon repeated exposures, are corrosive, cross-link living tissues and many synthetic materials, and may lose efficacy when pathogens enzymatically adapt to them. Alcohols are flammable and volatile and can be enzymatically degraded by certain bacterial pathogens. Quats are highly irritating to mucous membranes and over time can induce pathogen resistance, especially if they are not alternated with functionally different disinfectants. In contrast, peracetic acid (PAA), a potent oxidizer, liberates hydrogen peroxide (itself a disinfectant), biodegrades to carbon dioxide, water and oxygen, and is at least as efficacious as contact biocides e.g., halogens and aldehydes. Nevertheless, the standard form of liquid PAA is highly corrosive, is neutralized by metals and organic matter, gives off noxious odours and must be stored in vented containers. For the reasons stated above, Bioxy formulations were developed, a series of powder forms of PAA, which are odourless, stable in storage and safe to transport and handle. They generate up to 10% PAA *in situ* when dissolved in water. A 0.2% aqueous solution of Bioxy (equivalent to 200 ppm PAA) effected a 6.76 log reduction in Methicillin-resistant *Staphylococcus aureus* (MRSA) within 2 minutes after application. A 5% aqueous solution of Bioxy achieved a 3.93 log reduction in the bovine tuberculosis bacillus *Mycobacterium bovis*, within 10 minutes after contact. A 1% solution of Bioxy reduced vancomycin-resistant enterococci (VRE) and *Pseudomonas aeruginosa* by 6.31 and 7.18 logs, respectively, within 3 minutes after application. A 0.5% solution of Bioxy inactivated porcine epidemic diarrhea virus (PEDV) within 15 minutes of contact, and a 5% solution of Bioxy realized a 5.36 log reduction in the spores of *Clostridium difficile* within 10 minutes of application. In summary, Bioxy is safe and easy to transport and store, poses negligible human, animal and environmental health risks, shows high levels of pathogen control efficacy and does not induce microbial resistance. Further investigations are recommended to explore its use as an industrial biocide.

## Introduction

Several categories of surface disinfectants that have been used for decades in medical, veterinary, food processing and institutional applications include halogens (ex. bromine, hypochlorite and iodophors), quaternary ammonium compounds (“quats”) and aldehydes (ex. formaldehyde, glutaraldehyde and *o*-phthalaldehyde).

Halogens are highly electronegative Group 17 elements and potent oxidizers. It has been postulated that halogens kill microbial pathogens by reacting with the thiol groups of the cysteine and methionine residues in both their structural (membrane) and enzymatic proteins to form chloramines, resulting in membrane lysis due to loss of integrity and the destruction of enzyme function. In addition, halogens may saponify membrane lipids [1].

Chlorine is almost never used in its elemental form since this substance is highly toxic, corrosive and reactive. Instead, chlorine for sanitizing purposes is furnished in several compounded forms that store active chlorine in relatively stable forms and release it gradually. These include chlorine dioxide, chloramine and sodium dichloroisocyanurate. An extremely widely used form of chlorine is hypochlorite, either in liquid (sodium salt) or powder (calcium salt) form. Unfortunately, several metals (including stainless steel, chrome, aluminium, copper, brass and bronze) may be irreversibly corroded by even short-term repeated exposures to hypochlorite, and the resultant reduction of the disinfectant may liberate toxic chlorine gas. Moreover, certain species of mycobacteria (all of which are pathogenic to humans and/or livestock) are 100–300 times more resistant to hypochlorite than are most strains of *Escherchia coli* [2]. At least 10-minute exposure to hypochlorite may be required to kill the endospores of bacterial pathogens such as *Bacillus spp*. and *Clostridium spp*.

Iodine as a disinfectant is usually applied in the form of an iodophor which consists of polyvinylpyrrolidone (PVP) and elemental iodine. Rarely, iodine may be administered in the form of an ethanol solution of elemental iodine. Since it is the iodine *per se* that is biocidal, and since iodine is a halogen, the mode of action of an iodophor is very similar to that of hypochlorite. Nevertheless, iodine is a far weaker oxidizer than chlorine and hence longer contact times and higher concentrations of product are required to achieve efficacy levels with iodophors that are comparable to those of hypochlorite. The free iodine they release corrodes metals and permanently stains many materials. Furthermore, there is evidence to indicate that iodophors do not consistently penetrate bacterial glycocalyx biofilms [3]. This deficiency could result in only partial pathogen kill, which may permit surviving cells within the matrices to re-establish new colonies.

Quaternary ammonium compounds “Quats” are cationic surfactants with a generalized structure NR_4_^+^, wherein R is either an aryl- or an alkaryl group. Quats retain their positive charge regardless of ambient pH and are usually supplied in the form of halide salts. The biocidal activity of quats is attributed to their ability to bind irreversibly to the negatively charged moieties on the phospholipid and protein constituents of bacterial membranes [4]. Nevertheless, it is precisely this chemical property which may account for the fact that quats are reproductive toxicants in fish, and therefore, may be regarded as potential environmental contaminants [5]. It has been observed that multiple quat exposures reduced fecundity in mice by decreasing their litter sizes and increasing the rates of miscarriage and stillbirth in pregnant females [6]. In addition, quats are inactivated by hard water, anionic surfactants and acidity and demonstrate poor or limited efficacy against non-enveloped viruses, mycobacteria, bacterial endospores and molds [5].

Aldehydes are organic compounds with the general structure R—CHO, that is, a carbonyl group to which hydrogen and R-group moieties are bonded. Most aldehydes are volatile and can denature proteins by binding to the thiol groups in cysteine and methionine residues. Aldehydes can also bind to the nitrogen moieties of purine bases in nucleic acids [7]. Aldehydes commonly used as surface disinfectants include formaldehyde (which is also a tissue preservative), glutaraldehyde and *o*-phthalaldehyde. Aldehydes offer a wide spectrum of biocidal activity: due to their mode of action, they are lethal to bacterial and fungal spores, as well as viruses and mycobacteria. Nevertheless, certain microbial pathogens, specifically some *Mycobacterium* species, have already demonstrated resistance to aldehydes [8]. Chronic human exposures to aldehyde vapours can lead to asthma-like bronchospasms and repeated skin contact can induce severe dermatitis. The Occupational Safety and Health Administration (OSHA) has established a Permissible Exposure Limit (PEL) of 0.75 ppm formaldehyde in air, based upon an eight-hour Time-Weighted Average (TWA). OSHA has also set a Short-Term Exposure Limit (STEL) of 2 ppm formaldehyde over a fifteen-minute period [9].

Glutaraldehyde is a dialdehyde that has been used extensively as a veterinary virucide in animal husbandry. For example, glutaraldehyde effectively controls rotavirus in horses [10] and porcine epidemic diarrhea virus (PEDV) in pigs [11]. Glutaraldehyde efficacy is not compromised by the presence of organic matter, but it is greatly reduced by low temperature and short contact times [10], both of which are likely to occur in livestock management. In terms of human medicine, glutaraldehyde at a minimum concentration of 2.4% v/v has been used in the high-level disinfection of surgical and diagnostic instruments [7]. Glutaraldehyde also retards warts caused by human papillomavirus (HPV), and cauterizes the lesions formed by this virus [12]. However, recent studies have shown that glutaraldehyde, even at extended contact times, was unable to inactivate HPV [13, 14]. In fact, the only disinfectants that were able to inactivate HPV were strong oxidizers. Due to its mode of action, glutaraldehyde is a respiratory, dermal and ocular irritant, and is a skin sensitizer. The Organisation for Economic Cooperation and Development (OECD) has set a human glutaraldehyde exposure limit of 0.1–0.2 ppm [15].

Peroxy acids or peracids are inorganic- and organic compounds whose structure includes the –O-OH moiety. All of them are very strong oxidizers. Their principal biocidal mode of action is the oxidation of thiol moieties in the cysteine and methionine residues of both enzymatic and structural proteins. This reaction is generally instantaneous. Moreover, unlike hydrogen peroxide, peracids are resistant to degradation by catalase because their pH is far lower than those of peroxides and because they are too bulky to fit into the peroxide binding site on the enzyme; catalase will decompose hydrogen peroxide into water and oxygen.

The most extensively applied organic peroxy acid is peroxyacetic acid, also known as peracetic acid or simply PAA. This compound is produced industrially by the cobalt- or copper-catalyzed autoxidation of acetaldehyde and by the sulfuric acid-catalyzed reaction between acetic anhydride and concentrated hydrogen peroxide. Both processes involve the manipulation of highly corrosive and potentially toxic and combustible reagents. Liquid [PAA is itself a potent oxidizer (eV = 1.81; compared with hypochlorite whose eV = 1.36) [16], corrosive and malodorous, and it releases oxygen in storage which must be constantly vented so that the containers do not explode. Nevertheless, unlike hypochlorite, chlorine dioxide and other halogenated biocides, PAA is biodegradable. It decomposes to acetic acid, water and oxygen, and ultimately the acetic acid is broken down to carbon dioxide and water. Unfortunately, this degradation process is greatly enhanced in the presence of organic matter. Therefore, as is the case with most biocides, PAA application must be preceded by a cleaning step using a surfactant-based formulation in order to remove debris from the surface to be sanitized. In addition, PAA readily decomposes in the presence of base metal cations. Even excessively alkaline or hard water may shorten the shelf life of liquid PAA, so chelating agents such as dipicolinate or etidronate are often added to the formulation.

**Fig 1:**
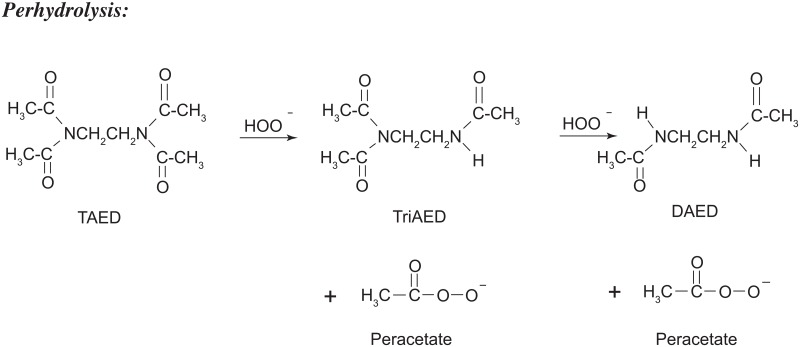
Peracetic acid generation *in situ* via the reaction between TAED and [hydrogen peroxide derived from] percarbonate [17].

Another method of synthesizing PAA involves the reaction between tetraacetylethylenediamine (TAED) and a peroxy compound such as perborate, percarbonate or persulfate. This technology was first developed for the laundry and textile industries as a means of incorporating nonchlorinated bleach into detergent powders which could liberate PAA *in situ*. A relatively minor component of this chemical technology was adopted for pulp bleaching in the paper, paperboard and cardboard manufacturing industry. Often the preferred peroxy compound is percarbonate, which is chemically similar to sodium carbonate, also known as washing soda, a water conditioner extensively used in the laundry industry. In this reaction, TAED donates two acetyl moieties to the hydrogen peroxide that is liberated from the percarbonate when it is in aqueous solution. Both of these reagents are in solid powder form, and peracetic acid generation occurs only when they are dissolved in water. Several advantages of this approach to PAA production over the conventional liquid reagent methods include the fact that (a) the powder product is relatively nonreactive and stable in storage and transport; (b) it is compact and requires no special containers; (c) it can generate up to ten percent PAA, and there is no need to dilute corrosive stock concentrates in order to prepare solutions of the desired target PAA concentration. Both the reagents (TAED, percarbonate) and the product (PAA) decompose into relatively innocuous and biodegradable or biotransformable by-products (sodium carbonate, ammonia, carbon dioxide, acetic acid, oxygen, water), so the environmental footprint of this formulation is minimal. The following Fig 1 is the reaction mechanism between TAED and percarbonate that generates PAA *in situ*:

The peroxide is generated from percarbonate as follows:

2Na2CO3•3H2O2→4Na + 2CO32− + 3H2O2

It is noteworthy to mention that other compounds such as titanium dioxide, silver nitrate and zinc oxide [18–22] have also strong bactericidal properties against various pathogens. The aim of this study was to assess the wide spectrum high biocidal potency of Bioxy formulation when dissolved in water at various concentrations in order to propose to end-users a safer alternative to traditional disinfectants.

## Materials and methods

### Bioxy vs. *Clostridium difficile* endospores

*Clostridium difficile* strain ATCC 43598 was grown for three days at 37°C on fructose-sheep blood-taurocholate (FA-ST) agar [protease peptone (HiMedia), sodium phosphate dibasic (Machron Chemicals, potassium phosphate monobasic (EMD Millipore), sodium chloride (Fisher Chemical), magnesium sulfate (Machron Chemicals), fructose (Fisher Chemical), sheep blood (Hemostat Labs), and sodium taurocholate (Alfa Aesar)] prepared from a culture grown for 3 d at 37°C. By this time, sporulation had occurred and spores were harvested by first killing vegetative cells by subjecting them to 70°C for 10 min, then centrifuging the suspension to form a spore pellet. The purified spores were suspended in sterile water used for high performance liquid chromatography (HPLC) then stored overnight at 4°C. This process was repeated 3 x. The spore suspension was inspected via phase-contrast microscopy and maintained at 4°C.

A 5.0% w/v Bioxy solution was prepared by analytically weighing out 50 g of the product in a sterile 2 L bottle followed by the addition of 950 mL sterile DI water to the bottle and then mixing for a minimum of 10 min.

A neutralizer solution consisting of a sterile mixture of 12.7% Tween 80 (Fisher BioReagents), 6.0% Tamol (Rohm and Haas), 1.7% lecithin (Sigma-Aldrich), 1.0% peptone (HiMedia), 1.0% cysteine (Spectrum) and 500 mM Tris buffer (pH 7.0, Fisher BioReagents) was added to 9 mL test tubes.

An aliquot of 9.9 mL disinfectant solution was added to each sterile 50 mL polypropylene centrifuge tube (Sarstedt). The tubes were then equilibrated to 20°C in a water bath. A 0.1 mL aliquot of *C*. *difficile* spores was then added to the disinfectant solution. After 10 min, 1.0 mL spore/disinfectant suspension was added to 9.0 mL neutralizer followed by thorough mixing. Two minutes later, a series of dilutions of the neutralized suspension was prepared using sterile 9 mL physiological saline solution (PSS) blanks.

Viable cell counts were determined using membrane filtration (Millipore manifold and Millipore disposable plastic funnels with a BioRad vacuum pump). Membrane filters (47 mm, 0.45 μm pore size, Millipore) were rinsed with 100 mL sterile PSS, inoculated in duplicate with 1 mL spore suspension aliquots, then transferred to FA-ST plates which were then incubated anaerobically for 3 d at 37°C with an ANOXOMAT system. Colonies on each filter were enumerated, then log reduction values and kill rates (%) were calculated.

Controls consisted of (a) spore suspension titers derived from membrane filtration assays on selected 1:10 PSS spore suspension dilutions, and (b) a neutralizer control prepared by inoculating a mixture of 9.0 mL neutralizer and 1.0 mL disinfectant solution with 0.1 mL of the 1:1x10^5^ titer dilution, incubating it for 20 min prior to dilution, then assaying it via membrane filtration of duplicate samples. This mixture resulted in a cell concentration of about 68 colony-forming units (CFU)/mL.

### Bioxy vs. *Pseudomonas aeruginosa* and Vancomycin-Resistant *Enterococcus* (VRE)

A 5 mL aliquot of *Pseudomonas aeruginosa* strain ATCC 15442 was grown for 24 h at 37°C in nutrient broth. The suspension was purified by centrifugation and the pellet was resuspended in 1 mL sterile physiological saline solution (PSS).

A 5 mL aliquot of Vancomycin-resistant *Enterococcus faecalis* (VRE) clinical strain 112105 was grown for 24 h at 37°C in nutrient broth (Becton-Dickinson). The suspension was purified by centrifugation and the pellet was resuspended in 0.5 mL sterile PSS.

A 1.0% w/v Bioxy solution was prepared by analytically weighing out 10 g of the product, then adding it to a sterile 2 L bottle, then adding 990 mL sterile DI water to the bottle, then mixing for a minimum of 10 min.

A neutralizer solution consisting of a sterile mixture of 12.7% Tween 80, 6.0% Tamol, 1.7% lecithin, 1.0% peptone, 1.0% cysteine and 500 mM Tris buffer was added to 9 mL test tubes.

An aliquot of 9.9 mL disinfectant solution was added to each sterile 50 mL polypropylene centrifuge tube and these were equilibrated to 20°C in a water bath. A 0.1 mL aliquot of *Pseudomonas aeruginosa* suspension or VRE suspension was then added to the disinfectant solution. After 3 min, 1.0 mL *Pseudomonas aeruginosa*/disinfectant suspension or VRE/disinfectant suspension was added to 9.0 mL neutralizer followed by thorough mixing. Two minutes later, a dilution series of the neutralized suspension was prepared using sterile 9 mL physiological saline solution (PSS) blanks.

Viable cell counts were determined using membrane filtration as described previously. Duplicate 1 mL aliquots were plated. Membrane filters were rinsed with 100 mL sterile PSS then transferred to Columbia agar (Becton-Dickinson) plates which were then incubated aerobically for 24–48 h at 37°C. Colonies on each filter were enumerated, then log reduction values and kill rates (%) were calculated.

Controls consisted of (a) titers derived from membrane filtration assays on selected 1:10 PSS suspension dilutions, and (b) a neutralizer control prepared by inoculating a mixture of 9.0 mL neutralizer and 1.0 mL disinfectant solution with 0.1 mL of the 1:1x10^5^ titer dilution, incubating it for 20 min prior to dilution, then assaying it via membrane filtration of duplicate samples. These mixtures resulted in cell concentrations of about 743 CFU/mL for *Pseudomonas aeruginosa* and 102 CFU/mL for VRE.

### Bioxy vs. *Mycobacterium bovis* (bovine tuberculosis)

*Mycobacterium bovis* strain ATCC 35743 was prepared from a frozen suspension grown in modified Proskauer-Beck medium [asparagine (Difco), magnesium sulphate (JT Baker), magnesium citrate (Sigma-Aldrich), and glycerol (Fisher)]. Thawed suspensions were mixed in a Teflon-on-glass tissue grinder (Wheaton) using equal volumes of phosphate-buffered gelatin solution [NaH_2_PO_4_ (Fisher), Na_2_HPO_4_·7H_2_O (Machron Chemicals), Bacto-Gelatin (Difco)] on ice, then homogenized 2 min, then diluted to 1:10 using PSS + 0.1% Tween 80 (Fisher BioReagents), then diluted to 1:5 using PSS + 0.1% Tween 80. The suspensions were vortexed and kept on ice until they were used to inoculate the disinfectants.

A 5.0% w/v Bioxy solution was prepared by analytically weighing out 50 g of the product, then adding it to a sterile 2 L bottle, then adding 950 mL sterile DI water to the bottle, then mixing for 30 min on a magnetic stirrer plate.

A neutralizer solution was prepared and it consisted of 12.7% Tween 80, 6.0% Tamol, 1.7% lecithin, 1.0% peptone and 1.0% cysteine.

An aliquot of 9.0 mL disinfectant solution was added to each sterile 50 mL polypropylene centrifuge tube and these were equilibrated to 20°C in a water bath. A 1.0 mL aliquot of *M*. *bovis* was then added to the disinfectant solution. At the 3-, 5-, 10- and 20-min marks, 1.0 mL aliquots of the *M*. *bovis* suspension were added to 9.0 mL neutralizer followed by vortexing. Two minutes later, a 1:10 dilution series of the neutralized suspension was prepared using sterile 9 mL physiological saline solution (PSS) blanks.

Viable cell counts were determined using membrane filtration as described previously. Duplicate 1 mL aliquots were plated. Membrane filters were rinsed with 100 mL sterile PSS then transferred to Mycobacteria 7H11 agar (Becton Dickinson) plates enriched with 10% oleic acid, albumin, dextrose, catalase (OADC, Becton Dickinson) which were then incubated in plastic bags at 37°C for about 21 d. Colonies on each filter were enumerated, then log reduction values and kill rates (%) were calculated.

Titers were prepared by aseptically transferring 1.0 mL of the culture medium to 9.0 mL PSS then vortexing the mixture. Thence, serial dilutions of 10^−1^ through 10^−7^ inclusive were made. In order to obtain cell densities, 1 mL aliquots from dilutions 10^−5^ through 10^−7^ were plated onto Mycobacteria 7H11 agar enriched with 10% OADC, then incubated in plastic bags at 37°C for about 21 d. Viable plate counts were then performed.

Static controls were prepared as follows: a 0.9 mL aliquot of a 5% Bioxy solution and 9 mL neutralizer mixture were aseptically added to a sterile test tube. The mixture was vortexed for 5 min. Then 0.1 mL mycobacterium culture was added to the test tube and the mixture was vortexed again. This mixture was then serially diluted from 10^−1^ through 10^−5^ inclusive. One mL aliquots each of dilutions 10^−3^ through 10^−5^ were filtered. The filters were then plated onto Mycobacteria 7H11 agar enriched with 10% OADC, then incubated in plastic bags at 37°C for about 21 d. Viable plate counts were then performed as described previously.

Neutralizer toxicity controls were prepared as follows: a 1 mL aliquot of the mycobacterium culture was combined with 9 mL room temperature PSS in a test tube. Then a 1 mL aliquot of the bacterial suspension/PSS mixture was added to 9 mL neutralizer and vortexed for 5 min. This mixture was then serially diluted from 10^−1^ through 10^−5^ inclusive. One mL aliquots each of dilutions 10^−3^ through 10^−5^ were filtered. The filters were then plated onto Mycobacteria 7H11 agar enriched with 10% OADC, then incubated in plastic bags at 37°C for about 21 d. Viable plate counts were then performed.

### Bioxy applied electrostatically vs. Methicillin-resistant *Staphylococcus aureus* (MRSA)

A 5 mL aliquot of Methicillin-resistant *Staphylococcus aureus* (MRSA) strain ATCC 43300 was grown for 24 h at 37°C in nutrient broth. The suspension was purified by centrifugation and the pellet was resuspended in 1 mL sterile physiological saline solution (PSS).

A 0.2% w/v solution of Bioxy was prepared by analytically weighing out 2.0 g then adding it to 998 mL sterile DI water in a sterile 2 L bottle. The solution was mixed on a magnetic stirrer plate for about 15 minutes prior to loading it into an electrostatic sprayer (Model SC-EB, Electrostatic Spraying Systems, Watkinsville, GA, U.S.A.). A positive cartridge was loaded into the sprayer and about 80 mL was collected from the sprayer in a sterile 500 mL bottle. The positive cartridge was then replaced with a negative cartridge and about 80 mL of the solution was collected as before into another sterile 500 mL bottle.

The neutralizer solution consisted of a sterile mixture of 12.7% Tween 80, 6.0%, 1.7% lecithin, 1.0% peptone and 1.0% cysteine. An aliquot of 9.9 mL disinfectant solution was added to each sterile 50 mL polypropylene centrifuge tube and these were equilibrated to 20°C in a water bath. A 0.1 mL aliquot of MRSA suspension was then added to the disinfectant solution. After 2 min, 1.0 mL MRSA/disinfectant suspension was added to 9.0 mL neutralizer followed by thorough mixing. Two minutes later, a dilution series of the neutralized suspension was prepared using sterile 9 mL physiological saline solution (PSS) blanks.

Viable cell counts were determined using membrane filtration as described previously. Duplicate 1 mL aliquots were plated. Membrane filters were rinsed with 100 mL sterile PSS then transferred to Columbia agar plates which were then incubated aerobically for 24–48 h at 37°C. Colonies on each filter were enumerated, then log reduction values and kill rates (%) were calculated.

Controls consisted of (a) titers derived from membrane filtration assays on selected 1:10 PSS suspension dilutions, and (b) a neutralizer control prepared by inoculating a mixture of 9.0 mL neutralizer and 1.0 mL disinfectant solution with 0.1 mL of the 1:1x10^5^ titer dilution, incubating it for 20 min prior to dilution, then assaying it via membrane filtration of duplicate samples. These mixtures resulted in cell concentrations of about 282 CFU/mL.

### Bioxy vs. porcine epidemic diarrhea virus (PEDV)

A 0.5% w/v solution of Bioxy was prepared by adding 5 g of the product to 1 L sterile water then waiting 10 min for product activation and PAA release. Twenty μL PEDV inoculum was applied to each of a series of steel discs and then left to air dry for 30 min at room temperature. [Parallel control discs to which sterile water was applied were also prepared.] Forty μL of the PAA solution was applied to each of the steel discs. Parallel control discs to which sterile water was applied were also prepared to serve as negative controls. From each steel disc, PEDV was eluted at 5, 10, 15, and 30 min into 0.4 mL elution buffer consisting of a 0.05M glycine solution in 3% beef extract adjusted to pH 7.2. Comparable elutions were performed on the control disks as well. Tenfold serial dilutions of the viral eluates were prepared in Minimum Essential Medium (MEM) with Earle's Salts supplemented with L-glutamine, antibiotics, 10% TPB (tryptone phosphate broth) and 4.5 μg/ml trypsin. One hundred μL of appropriate dilutions were added to Vero-76 African green monkey kidney cells in 96-well plates. The plates were incubated in a 5% CO_2_ atmosphere at 37°C for 5, 10, 15, and 30 min, then stained by indirect fluorescence antibody (IFA; immunofluorescence) assay and the virus titers were calculated. Reductions in virus titers were calculated by comparing the differences between virus titers in the control discs and PAA-treated discs at 15, and 30 min.

### Bioxy efficacy as hard surface disinfectant vs. 3 human bacterial pathogens

Forty-eight-hour-old nutrient broth subcultures of the following microbial species were prepared: *Staphylococcus aureus* ATCC 6538, *Pseudomonas aeruginosa* ATCC 15442 and *Salmonella enterica* ATCC 10708. Twenty-four stainless steel carriers were inoculated with each test organism for a total of 72 carriers. A 2% w/v solution of Bioxy was prepared by adding 20 g to 995 mL sterile water in a 2 L bottle, then the contents were stirred 5 min. The solutions were then left standing for 5 min to activate the PAA fully. The PAA solution was dispensed as 10 mL aliquots in sterile test tubes then equilibrated to 20°C in a water bath. One bacteria-impregnated carrier was then aseptically transferred to each test tube in the water bath and the contents agitated to homogenize them. After 10 min contact time, the carriers were aseptically removed from each test tube and transferred to sterile neutralizer broth (Letheen medium + Tween 20) followed by shaking to blend the contents. All tubes were incubated at 36°C for 48 h. Media were scored + for growth if they became turbid and–(0 growth) if they remained clear. Sterility controls consisted of uninoculated aseptic carriers added to the disinfectant solution, left in it for 10 min, aseptically transferred to the sterile neutralizer broth then incubated at 36°C for 48 h. Neutralizer efficacy and toxicity were assayed by first aseptically transferring a sterile carrier to 10 mL disinfectant solution, then removing the carrier to neutralizer broth containing ≤100 CFU microbial pathogen, then incubating the media at 36°C for 48 h. Mean CFU/dry carrier were determined for each microorganism by placing each of 3 replicate carriers in 10 mL Letheen broth then mixing each thoroughly. The Letheen broths were serially diluted in sterile phosphate buffer dilution water (PBDW) blanks. Duplicate 1-mL aliquots were drawn from each dilution, plated onto tryptic soy agar and incubated at 36°C for 48 h. For viability controls, two inoculated carriers for each test microbe were placed in neutralizer recovery broth and incubated at 36°C for 48 h. Aliquots or colonies from all positive-growth media and plates were streaked onto [tryptic soy] agar plates and incubated at 36°C for 24 h. Sub-cultured colonies were Gram-stained and tested for biochemical markers specific to each microbial pathogen.

## Results

### Bioxy vs. *Clostridium difficile* endospores

It was determined that the 1:10^7^ dilution of the *C*. *difficile* stock suspension yielded the most reliable and accurate viable counts. For the duplicate assays, the numbers were 77 and 60 CFU/mL respectively. Back calculating, it was therefore obtained stock culture cell densities of 7.7E8 CFU/mL and 6E8 CFU/mL respectively, and the average was 6.85E8 CFU/mL as illustrated in Tables 1 and 2.

**Table 1:** Viable counts after 10 min exposure to 5% w/v Bioxy.

**Viable Counts After 10 min**	**Replicate**	**Stock suspension dilution**			
		1:10	1:100	1:1000	1:10,000
		(~7E7 CFU/mL)	(~7E6 CFU/mL)	(~7E5 CFU/mL)	(~7E4 CFU/mL)
	A	4	1	0	0
	B	2	0	0	0

**Table 2:** Control assay after 10 min exposure to 5% w/v Bioxy in neutralizer.

1:10^7^ dilution	A	B	Average	% of expected
**Expected count**	68		N/A	
**Actual counts**	65	56	61	89.7

Zero bacterial counts were obtained for the sterile pre-trial samples of the PSS, the 5% w/v Bioxy, the uninoculated culture media and the neutralizer mixture.

Log reduction (LR) and percent kill (PK) were determined as follows:

LR = −Log(S/So)

PK = (1 − (S/So)) × 100

where S = concentration of viable organisms after 10 min

where S_o_ = concentration of viable organisms at time 0

Based upon the data, it was found that log reduction was 5.36 and percent kill was 99.99956%.

### Bioxy vs. *Pseudomonas aeruginosa* and Vancomycin-Resistant *Enterococcus* (VRE)

It was determined that the 1:10^8^ dilution of the *P*. *aeruginosa* stock suspension yielded the most reliable and accurate viable counts. For the duplicate assays, the numbers were 66 and 84 CFU/mL respectively. Back calculating, we therefore obtained stock culture cell densities of 6.6E8 CFU/mL and 8.4E9 CFU/mL respectively, and the average was 7.5E9 CFU/mL as illustrated in Tables 3 and 4.

**Table 3:** Viable counts of *P*. *aeruginosa* after 3 min exposure to 1% w/v Bioxy.

**Viable Counts After 3 min**	**Replicate**	**Stock suspension dilution**		
		1:10	1:100	1:1000
		(~7.5E8 CFU/mL)	(~7.5E7 CFU/mL)	(~7.5E6 CFU/mL)
	A	0	0	0
	B	0	0	0

**Table 4:** *P*. *aeruginosa* control assay after 3 min exposure to 1% w/v Bioxy in neutralizer.

1:10^7^ dilution	A	B	Average	% of expected
**Expected count**	74		N/A	
**Actual counts**	30	42	36	48.7

Zero bacterial counts were obtained for the sterile pre-trial samples of both PSS, the 1% w/v Bioxy, the uninoculated culture media, the neutralizer mixture and the Columbia agar.

Log reduction (LR) and percent kill (PK) were determined as follows:

LR = −Log(S/So)

PK = (1 − (S/So)) × 100

where S = concentration of viable organisms after 3 min

where S_o_ = concentration of viable organisms at time 0

Based upon the data, it was found that for the *P*. *aeruginosa* assay log reduction was >7.18 and percent kill was >99.999993%.

It was determined that the 1:10^7^ dilution of the Vancomycin-resistant *Enterococcus faecalis* (VRE) stock suspension yielded the most reliable and accurate viable counts. For the duplicate assays, the numbers were 105 and 100 CFU/mL respectively. Back calculating, we therefore obtained stock culture cell densities of 10.5E8 CFU/mL and 10E8 CFU/mL respectively, and the average was 10.25E8 CFU/mL as illustrated in Tables 5 and 6.

**Table 5:** Viable counts of Vancomycin-Resistant *Enterococcus faecalis* (VRE) after 3 min exposure to 1% w/v Bioxy.

**Viable Counts After 3 min**	**Replicate**	**Stock suspension dilution**		
		1:10	1:100	1:1000
		(~10.25E7 CFU/mL)	(~10.25E6 CFU/mL)	(~10.25E5 CFU/mL)
	A	0	0	0
	B	0	0	0

**Table 6:** Vancomycin-Resistant *Enterococcus faecalis* (VRE) control assay after 3 min exposure to 1% w/v Bioxy in neutralizer.

Undiluted	A	B	Average	% of expected
**Expected count**	102		N/A	
**Actual counts**	96	108	102	100

Zero bacterial counts were obtained for the sterile pre-trial samples of both PSS, the 1% w/v Bioxy, the uninoculated culture media, the neutralizer mixture and the Columbia agar.

Log reduction (LR) and percent kill (PK) were determined as follows:

LR = −Log(S/So)

PK = (1 − (S/So)) × 100

where S = concentration of viable organisms after 3 min

where S_o_ = concentration of viable organisms at time 0

Based upon the data, it was found that for the Vancomycin-resistant *Enterococcus faecalis* (VRE) assay log reduction was >6.31 and percent kill was >99.999951%.

### Bioxy vs. *Mycobacterium bovis*

It was determined that the 1:10^5^ dilution of the *M*. *bovis* stock suspension yielded the most reliable and accurate viable counts. For the duplicate assays, the numbers were 10 and 7 CFU/mL respectively. Back calculating, we therefore obtained stock culture cell densities of 1E6 CFU/mL and 7E5 CFU/mL respectively, and the average was 8.5E5 CFU/mL as illustrated in Tables 7 and 8. *The M*. *bovis* static control assay after 5 min exposure to neutralizer alone is illustrated in Table 9.

**Table 7:** Viable counts of *M*. *bovis* after different exposure times to 5% w/v Bioxy.

Contact time (min)	Replicate	Stock suspension dilution		
		1:10	1:100	1:1000
		(~8.5E3 CFU/mL)	(~8.5E2 CFU/mL)	(~8.5E1 CFU/mL)
**3**	A	TNTC	66	5
**3**	B	TNTC	77	6
**5**	A	79	8	1
**5**	B	106	8	1
**10**	A	1	0	0
**10**	B	1	1	0
**20**	A	0	0	0
**20**	B	0	0	0

**Table 8:** *M*. *bovis* neutralizer toxicity control assay after 5 min exposure to neutralizer in PSS.

**Viable Counts After 5 min**	**Replicate**	**Stock suspension dilution**		
		1:1000	1:10000	1:100000
		(~8.5 CFU/mL)	(~0.85 CFU/mL)	(~0.085 CFU/mL)
	A	12	2	0
	B	22	1	0

**Table 9:** *M*. *bovis* static control assay after 5 min exposure to neutralizer alone.

**Viable Counts After 5 min**	**Replicate**	**Stock suspension dilution**		
		1:1000	1:10000	1:100000
		(~8.4 CFU/mL)	(~0.84 CFU/mL)	(~0.084 CFU/mL)
	A	20	1	0
	B	27	1	0

Zero bacterial counts were obtained for the sterile pre-trial samples of the PSS, the 5% w/v Bioxy, the uninoculated culture media, the neutralizer mixture and the 7H11 agar. Neither the PSS + 0.1% Tween 80 nor the phosphate buffered gelatin was assayed.

Log reduction (LR) and percent kill (PK) were determined as follows:

LR = −Log(S/So)

PK = (1 − (S/So)) × 100

where S = concentration of viable organisms after 3, 5, 10 and 20 min

where S_o_ = concentration of viable organisms at time 0

The efficacy of Bioxy at 5% w/v concentration against *M*. *bovis* is illustrated in Table 10.

**Table 10:** *M*. *bovis* log reduction (LR) and percent kill (PK) in response to various exposure times to 5% w/v Bioxy.

Contact time (min)	3	5	10	20
**LR**	1.08	1.96	3.93	>4.23
**PK**	91.6	98.9	99.988	>99.994

### Bioxy vs. Methicillin-resistant *Staphylococcus aureus* (MRSA)

It was determined that the 1:10^8^ dilution of the Methicillin-resistant *Staphylococcus aureus* (MRSA) stock suspension yielded the most reliable and accurate viable counts. For the duplicate assays, the numbers were 29 and 26 CFU/mL respectively. Back calculating, we therefore obtained stock culture cell densities of 29E8 CFU/mL and 26E8 CFU/mL respectively, and the average was 27.5E8 CFU/mL as illustrated in Tables 11 and 12.

**Table 11:** Viable counts of Methicillin-resistant *Staphylococcus aureus* (MRSA) after 2 min exposure to positively- and negatively charged 0.2% w/v Bioxy.

**Viable Counts After 2 min**	**Replicate**	**Stock suspension dilution**					
		1:10		1:100		1:1000	
		(~27.5E5 CFU/mL)		(~27.5E4 CFU/mL)		(~27.5E3 CFU/mL)	
		+ charge	- charge	+ charge	- charge	+ charge	- charge
	A	0	0	0	0	0	0
	B	0	0	0	0	0	0

**Table 12:** Methicillin-resistant *Staphylococcus aureus* (MRSA) control assay after 2 min exposure to positively- and negatively charged 0.2% w/v Bioxy in neutralizer.

1:10 dilution	A	B	Average	% of expected
**Expected count**	28		N/A	
**Actual counts (+ charge)**	24	23	23.5	95
**Actual counts (- charge)**	28	33	30.5	106

Zero bacterial counts were obtained for the sterile pre-trial samples of both PSS, both 0.2% w/v Bioxy solutions, the neutralizer mixture and the Columbia agar.

Log reduction (LR) and percent kill (PK) were determined as follows:

LR = −Log(S/So)

PK = (1 − (S/So)) × 100

where S = concentration of viable organisms after 2 min

where S_o_ = concentration of viable organisms at time 0

Based upon the data, it was found that for the Methicillin-resistant *Staphylococcus aureus* (MRSA) assay, log reduction was >6.76 and percent kill was 99.999982% after 2 min exposure to both positively- and negatively charged 0.2% w/v Bioxy.

### Bioxy vs. porcine epidemic diarrhea virus (PEDV)

The efficacy of Bioxy at 0.5% w/v concentration against PEDV at 15 min contact time is illustrated in Table 13.

**Table 13:** PEDV control efficacy of 0.5% w/v Bioxy.

Contact time (min)	Control (untreated) virus suspension titer*	Post-treatment virus suspension titer*	% virus reduction relative to control
**15**	320	<1	>99.99
**30**	320	<1	>99.99

*TCID_50_/mL, where TCID = median tissue culture infective dose, and where 1 TCID_50_/mL ≈ 0.7 PFU [plaque-forming unit]/mL

### Bioxy efficacy as hard surface disinfectant vs. 3 human bacterial pathogens

Bioxy solution at 2% (w/v) killed *Staphylococcus aureus*, *Salmonella enterica* and *Pseudomonas aeruginosa* in 10-min contact time in 60 growing treated carriers as shown in Tables 14 to 16.

**Table 14:** Disinfection results against *Staphylococcus aureus*.

Sample	Challenge organism	Exposure time	# of treated carriers	# of growing treated carriers	Growth results
Bioxy 2% solution (w/v)	*Staphylococcus aureus* ATCC # 6538	10 min	60	0	0/60
Bioxy 2% solution (w/v)	*Staphylococcus aureus* ATCC # 6538	10 min	60	0	0/60
Bioxy 2% solution (w/v)	*Staphylococcus aureus* ATCC # 6538	10 min	60	0	0/60

**Table 15:** Disinfection results against *Salmonella enterica*.

Sample	Challenge organism	Exposure time	# of treated carriers	# of growing treated carriers	Growth results
Bioxy 2% solution (w/v)	*Salmonella enterica* ATCC # 10708	10 min	60	0	0/60
Bioxy 2% solution (w/v)	*Salmonella enterica* ATCC # 10708	10 min	60	0	0/60
Bioxy 2% solution (w/v)	*Salmonella enterica* ATCC # 10708	10 min	60	0	0/60

**Table 16:** Disinfection results against *Pseudomonas aeruginosa*.

Sample	Challenge organism	Exposure time	# of treated carriers	# of growing treated carriers	Growth results
Bioxy 2% solution (w/v)	*Pseudomonas aeruginosa* ATCC # 15442	10 min	60	0	0/60
Bioxy 2% solution (w/v)	*Pseudomonas aeruginosa* ATCC # 15442	10 min	60	0	0/60
Bioxy 2% solution (w/v)	*Pseudomonas aeruginosa* ATCC # 15442	10 min	60	0	0/60

## Discussion

Following are some of the criteria which must be considered when investing in disinfection and sterilization technologies for the food processing industry, human medicine, veterinary medicine and institutional applications: (a) product and application equipment costs; (b) product consumption volumes; (c) product application frequencies; (d) product application learning curves; (e) ease and safety of product handling and use, and personal protection equipment required, if any; (f) post-application downtimes; (g) post-treatment rinse requirements, if any; (h) product storage requirements; (i) waste disposal methods; (j) product shelf life; (k) product environmental impact and biodegradability; (l) risk of pathogen resistance to product; (m) product efficacy relative to competing technologies; (n) risk of food contamination by product; (o) availability of regulatory approvals for intended product use.

Peracetic acid (PAA), also known as peroxyacetic acid and ethaneperoxoic acid, was first patented in 1950 for the post-harvest disinfection of raw fruits and vegetables destined for consumption without further processing [23]. The United States Environmental Protection Agency (USEPA) subsequently approved the product for this intended application.

Commercial liquid PAA is manufactured either by the cobalt-or copper-catalyzed oxidation of acetaldehyde [24] or by the oxidative reaction of acetic anhydride or glacial acetic acid with concentrated hydrogen peroxide, usually catalyzed by sulfuric acid [25]. Since the maximum available concentration of the hydrogen peroxide precursor is 30% v/v, the strongest industrially available PAA is rated at about 35% v/v. At this concentration, liquid PAA is considered a hazardous material according to most government health, safety and environmental protection agencies. PAA spontaneously decomposes into acetic acid, oxygen and water, and this degradation process is greatly accelerated by exposures to UV light, air, heat and base metals and, to a lesser extent, organic matter. In order to prolong PAA shelf life, sequestering agents may be added to it. These include dipicolinic- and etidronic acids. Although they will confer some protection of the PAA against metal cations in the water used to dilute it, they have no protective effect against organic debris or destructive physical factors.

Although the biocidal mode of action of PAA is not fully understood, it is believed that it functions in much the same way as other strong oxidizers like ethylene oxide, ozone and sodium hypochlorite via denaturation of enzymes and membrane- and structural proteins by binding to the thiol moieties on cysteine and methionine residues [7]. The oxidative reaction between thiols and PAA is instantaneous. Furthermore, PAA may also oxidize and irreversibly denature nucleic acids. For these reasons, it is likely that PAA prevents the pathogens from mutating and developing resistance against it. Therefore, development of microbial resistance to PAA is highly improbable. Nevertheless, endospores and biofilms, two protective barriers produced by many bacterial pathogens, could conceivably confer non-genetic resistance to PAA. Yet even a 0.2% v/v aqueous PAA solution effected a percent kill of 99.9% in three different strains of sporulating *Bacillus subtilis* within about 0.5–1.5 min of contact with the biocide [26]. Biofilms are composed of extracellular secretions of polysaccharides, which confer protection of vegetative bacterial cells against desiccation, predation and toxicants. Biofilms may also exclude oxygen and, therefore, increase the likelihood of survival of obligately anaerobic pathogens, in addition to the fact that many of these organisms can also produce endospores. Although one particular bacterial species may predominate in the matrix, it is quite rare to encounter a monoculture in natural biofilms. Generally, the bacterial populations in them consist of consortia of often unrelated species. For example, bacterial biofilms in food processing plants may contain representative species of *Listeria*, *Pseudomonas*, *Campylobacter*, *E*. *coli* and *Salmonella*. It has been shown that combinations of peracetic acid and hydrogen peroxide are more effective at killing bacterial consortium populations in synthetic biofilms than hypochlorites, quats, alcohols, organic acids, esters, aldehydes and silver compounds. The only exception was *Listeria;* no biocide, including the HP-PAA combinations, was adequately effective at penetrating biofilms created by this organism [27].

The fabrication, packaging, transport, storage and handling of liquid peracetic acid all pose hazards and risks in terms of worker safety. When PAA is manufactured via the cobalt- or copper-catalyzed oxidation of acetaldehyde, there is a risk of combustion of acetaldehyde vapours. Moreover, the PAA fumes themselves are malodorous, lachrymatory and toxic, causing respiratory, dermal and ocular irritation as well as cross-linkage of membrane proteins. The production of PAA using glacial acetic acid and concentrated hydrogen peroxide catalyzed by sulfuric acid involves the manipulation of three highly corrosive substances. In addition, hydrogen peroxide is a potent oxidizer, and acetic acid being relatively volatile (vapour pressure 11 m Hg at 20°C; evaporation rate 0.97 [relative to butyl acetate, whose evaporation rate = 1]), releases corrosive fumes which are highly irritating to mucous membranes, skin and eyes. Furthermore, the vapour is very dense (2.1; relative to air, whose density = 1), so the fumes would displace air and sink towards ground level [28].

The peracetic acid end product must be protected from degradation by base metal cations (period 4, d-block elements) present in the water used to dilute it. To this end, sequestering agents such as dipicolinic- or etidronic acid are added to the formulation. Even the high calcium- and/or magnesium levels in very hard water can reduce the activity and efficacy of peracetic acid.

Even under ideal storage conditions, liquid peracetic acid spontaneously decomposes into vinegar (dilute acetic acid) and continuously releases oxygen. For this reason, liquid PAA must be stored in vented containers to prevent the buildup of a potentially dangerous gas pressure in the headspace above the liquid.

A more convenient, cost effective and safer alternative to liquid PAA is a powder formulation that generates PAA *in situ* when it is dissolved in water. The reaction occurs between two granular products; the acetyl donor tetraacetylethylenediamine (TAED) and a solid peroxide generator such as sodium percarbonate. This technology has been incorporated into nonchlorinated bleaching powder laundry detergents since the 1950s, but here we adopt this formulation to provide PAA for hard surface disinfection on demand. This technology generates PAA that is chemically identical to that found in liquid disinfectant products. Provided that the powder PAA precursor is protected from atmospheric moisture in storage, its stability can be guaranteed for as long as five years. By contrast, liquid PAA may only remain stable for up to one year in storage. Even before then, it would be advisable to perform at least an iodometric titration on the product to determine its actual PAA concentration at the time of application. This step is not usually required for the powder PAA formulation that has been properly stored.

A very high efficacy levels of Bioxy was observed against vegetative *Pseudomonas aeruginosa*, VRE and MRSA cells (7.18, 6.31 and >6.76 log reductions respectively). In the *P*. *aeruginosa*/VRE study, a 1% w/v Bioxy solution was applied for 3 min; for the MRSA trial, 0.2% w/v Bioxy solution was used with a 2 min contact time. In future research, it is recommended to perform similar tests comparing Bioxy efficacy against that of chlorinated powders. It was shown that a 5% w/v solution of Bioxy effected a 5.36 log reduction in *Clostridium difficile* spores after a 10 min contact time. Moreover, Bioxy solution at 2% (w/v) killed *Staphylococcus aureus*, *Salmonella enterica* and *Pseudomonas aeruginosa* in 10-min contact time in 60 growing treated carriers. The efficacy of Bioxy at 0.5% w/v concentration against PED Virus at 15 min contact time was superior than 99.99% in terms of percent virus reduction relative to control.

Disinfectants that have demonstrated potent virucidal activity include PAA, glutaraldehyde and quats. A 1500 ppm liquid PAA solution effected a 5 log reduction in porcine parvovirus titers whereas even a 2500 ppm dilution of glutaraldehyde achieved only a 4 log reduction in the titers of the same pathogen [29]. The authors observed that in general the parvoviruses, whether bovine, canine, murine or porcine, were more susceptible to the biocidal action of PAA than were the other viral taxa they tested (specifically adenoviruses, noroviruses, rotaviruses), and were more resistant to glutaraldehyde than PAA.

In practice, there are inherent health and safety risks to workers handling both liquid PAA and glutaraldehyde. PAA mists and glutaraldehyde vapors are dermal, ocular and respiratory irritants. In environments and workplace settings where there is inadequate ventilation and/or deficient personal protection equipment, chronic exposures to these virucidal agents may result in multiple intoxication symptoms. Since solutions of Bioxy are nonvolatile and noncorrosive, these health hazards would be significantly reduced without sacrificing virucidal efficacy if Bioxy were applied in place of the liquid disinfectants.

Eleraky, Potgieter and Kennedy [30] contrasted the biocidal potencies of chlorine (as chlorine dioxide and as hypochlorite), quaternary ammonium compounds and a peroxy compound (peroxymonosulfate) against feline calicivirus and parvovirus. Whereas the chlorines (≈5,000 ppm) and the peroxy salt (1% w/v) effected 4 log- and 6 log reductions in the parvovirus and calicivirus titers, respectively, after a 10 min contact time, quats (≈1% v/v) achieved only 1 log- and 2 log viral titer reductions for calicivirus and parvovirus respectively. Moreover, once again there are worker safety issues involved in the use of the traditional disinfectant in this case: in the presence of heavy organic loads, even powder chlorine biocides such as dichloroisocyanurate and trichloro-*s*-triazinetrione decompose rapidly, lose their virucidal efficacy and release toxic chlorine gas. Quaternary ammonium compounds, when applied as aerosols, can severely irritate mucous membranes like most surfactants. In addition, quats have been shown to be reproductive toxicants to mammals and fish in long-term or chronic exposures [6]. Quats are also rapidly inactivated by soaps and organic detritus, and they have no efficacy against food-borne viruses such as norovirus [30]. In contrast, solutions of peroxy compounds such as Bioxy do not lose potency in the presence of typical soap residue or organic debris burdens, and in aerosol form these products rapidly dissociate into acetic acid, hydrogen peroxide, water and oxygen.

## Conclusions

Powder PAA formulations should be considered as viable alternatives to liquid PAA and other peroxides, both liquid- and powder chlorinated products, quats, aldehydes, iodophors, and alcohols, because they:

1. have been demonstrated repeatedly to provide a level of biocidal efficacy at least comparable to those of other disinfectant classes;
2. are compact, are not classified as dangerous goods for transportation purposes, do not require special containers or storage conditions and can be safely handled without specialized HazMat attire or PPE;
3. generate the active ingredient (PAA) *in situ*, at full strength, on demand, consistently, and in noncorrosive, nonvolatile, relatively odour-free solutions;
4. provide a nearly instantaneous contact pathogen kill at a very high level of efficacy; and
5. do not induce pathogen resistance, and in fact could be applied in resistance management by being used as an alternative to aldehydes, chlorines, iodophors, quats, etc.

Nevertheless, further research and development are required in order to determine how to activate the PAA more rapidly. In addition, more comparative studies are required, testing the microbicidal effectiveness of Bioxy relative to all of the aforementioned disinfectant classes.
